# Sedated and unsedated gastroscopy has no influence on the outcomes of patients with gastric cancer: a retrospective study

**DOI:** 10.1186/s12885-024-13413-0

**Published:** 2025-01-06

**Authors:** Chengke Yin, Yiwu Sun, Jie Liang, Xin Sui, Zhaoyi He, Ailing Song, Wenjia Xu, Lei Zhang, Yufei Sun, Jingshun Zhao, Fei Han

**Affiliations:** 1 Department of Anesthesiology, Harbin Medical University Cancer Hospital, 150 Haping Rd, Nangang District, Harbin, Heilongjiang 150081 China; 2 Department of Anesthesiology, Dazhou Central Hospital, 56 Nanyuemiao Rd, Tongchuan District, Dazhou, Sichuan 635000 China; 3 Department of Anesthesiology, Shanghai Jiaotong University First People’s Hospital, 85 Wujin Rd, Hongkou District, Shanghai, 200080 China

**Keywords:** Anesthesia, Gastric cancer, Sedated gastroscopy, Unsedated gastroscopy, Comfort of gastroscopy

## Abstract

**Background:**

Different anesthetic drugs and techniques may affect survival outcomes for gastric cancer (GC) after surgery. In this study, we investigated the association between sedated and unsedated gastroscopy on survival outcomes in patients with GC after surgery.

**Methods:**

This was a retrospective study of patients who were diagnosed with GC by gastroscopy and underwent gastrectomy from January 2013 to December 2017. They were grouped based on the examination modality: propofol-based sedated gastroscopy or unsedated gastroscopy. Propensity score matching (PSM) was used to balance the baseline variables. Survival outcomes and distant metastases were compared between these two groups.

**Results:**

Finally, 673 patients were enrolled, 160 in the sedated gastroscopy group and 513 in the unsedated gastroscopy group. After PSM, there were 160 patients in each group. There was no significant difference in overall survival outcomes in the sedated gastroscopy group compared to the unsedated gastroscopy group before PSM (HR = 0.761, 95% CI: 0.531–1.091, *P* = 0.139) or after PSM (HR = 0.874, 95% CI: 0.564–1.355, *P* = 0.547). There was no significant difference in the incidence of distant metastases between the two groups before PSM (16.9% vs. 20.7%, *P* = 0.294) or after PSM (16.9% vs. 23.8%, *P* = 0.126). To confirm that our patients behaved similarly to other studies, we performed a multivariate analysis and the results showed that sex, pathological TNM stage, Borrmann type, adjuvant treatment, and surgical resection range were all independent factors affecting survival outcomes in our patients.

**Conclusion:**

Our results showed no significant difference in the effects of sedated gastroscopy vs. unsedated gastroscopy on survival outcomes or distant metastases of patients after gastrectomy for GC.

## Background

Gastric cancer (GC) is the fifth most newly diagnosed cancer and the third leading cause of cancer death in the world, killing more than 800,000 people in 2018, and it remains a substantial contributor to the global cancer burden [1]. Surgical resection is the most effective way to remove the primary tumor and metastatic lymph nodes. However, the stress response and immunosuppressive effects caused by surgery have been suggested to possibly promote cancer cell migration, affecting the long-term survival outcome of patients [2, 3]. Anesthesia is a necessary component of cancer surgery, and there is growing concern that it may promote cancer recurrence or metastasis by affecting immune function and tumor cell biology [4, 5]. Recent studies have shown that patients with rectal or ovarian cancer who are put under propofol-based total intravenous anesthesia have better survival outcomes than those put under desflurane-based inhalational anesthesia [6, 7]. The impact of anesthesia on long-term survival outcomes in oncology patients remains controversial, as conflicting survival outcomes have been reported in patients with GC based on propofol-based total intravenous and inhalation anesthesia [8, 9]. In addition to undergoing anesthesia during surgical procedures, patients diagnosed with GC frequently received anesthetics during gastroscopy. Research on the effectiveness of sedated gastroscopy compared to unsedated gastroscopy has primarily focused on investigating the safety and efficacy of using anesthetics during the examination process [10, 11]. Propofol was widely used in sedated gastroscopy [12]. However, the impact of small and multiple episodes of administrations of propofol sedation and non-sedation techniques on the survival prognosis of patients with GC remains unclear.

Like surgical resection, diagnostic or palliative interventions have been associated with survival outcomes in patients with GC [13]. Gastroscopy can determine the location of gastric tumors and their macroscopic types, and biopsy tissue specimens can be taken under gastroscopy for pathological confirmation, making gastroscopy an important tool for diagnosing GC as well as for postsurgical review. Although gastroscopy can be performed without sedation, patients undergoing the examination are not as comfortable and often have a range of adverse effects, such as pain, nausea, vomiting and coughing [14]. These adverse reactions not only make people have a negative attitude toward unsedated gastroscopy but also reduce the quality of the examination and hinder the diagnosis and treatment of the disease [15]. Sedated gastroscopy has been widely accepted because it reduces patient anxiety and improves patient comfort and willingness to repeat the examination [16]. Gastroscopy is performed throughout the perioperative period and postsurgical review of patients with gastric cancer, which means that patients with a preference for sedated gastroscopy will undergo multiple sedation. To our knowledge, current studies on the effects of anesthetic techniques or anesthetics on long-term survival in cancer patients have focused on the surgical period and have not explored the effects of sedation taken for diagnostic or palliative interventions on long-term survival. Therefore, we conducted a retrospective study to assess whether sedated or unsedated gastroscopy had an impact on survival outcomes in patients with GC.

## Methods

### Participants and data sources

This study met the ethical standards of the Declaration of Helsinki and was approved by the Ethics Committee of Harbin Medical University Cancer Hospital (Approval Number: KY2022-36). This retrospective study focused on medical record information relevant to this study and did not impact patient safety or the treatment process. The permission conditions of the original informed consent were met, and the written consent of the subjects was obtained to allow the use of their medical records for this research project. In addition, the data collected were not related to personal information and were provided only to the investigator to ensure that no personal information of the patient was disclosed. Patient records and personal information were deidentified prior to statistical analysis.

From January 2013 to December 2017, 850 patients were diagnosed with GC by gastroscopy and underwent gastrectomy. The entire treatment process of selected patients was carried out in Harbin Medical University Cancer Hospital, including all stages of diagnosis, treatment and review. The exclusion criteria were inoperable distant metastases, coexistence of other primary cancers, preoperative adjuvant therapy and incomplete medical records.

### Anesthetic techniques

All patients were informed of the differences between sedated and unsedated gastroscopy, including the benefits, risks, limitations, and possible alternatives, and voluntarily signed an informed consent form. All patients underwent a history and physical examination prior to gastroscopy, with special emphasis on sedation-related issues, and the details of the examination followed the published guidelines of the American Society of Anesthesiologists for anesthesia assistance during gastrointestinal endoscopy [17, 18]. All patients received oral administration of dacronine hydrochloride to reduce throat discomfort prior to gastroscopy. In the unsedated gastroscopy group, no sedated anesthetic was used, so they underwent the examination by the endoscopist in a conscious state. In the sedated gastroscopy group, patients had to fast for at least 2 h since consuming clear liquids and at least 6 h since consuming light foods. In the operating room, the intravenous access was opened and secured, and 3 L/min of oxygen ventilation was administered via the nasal catheter. Patients’ vital signs were regularly monitored by recording hemoglobin oxygen saturation, noninvasive blood pressure, and electrocardiogram. Individuals respond differently to sedation, which may be influenced by the patient’s health status, age, preoperative anxiety, and pain tolerance, so patients may require varying degrees of sedation during such an examination [19]. The anesthesiologist administered propofol intravenously to induce and maintain anesthesia at an initial dose of 1.5-2 mg/kg. Gastroscopy was not started until the patient lost consciousness, the eyelash reflex disappeared, and breathing was stable. Then, 30 to 40 mg of propofol was repeated to maintain adequate sedation. The patient was not intubated during the sedation process. Trained anesthesiologists were dedicated to the uninterrupted monitoring of the patient’s clinical and physiological parameters throughout the examination. They were also qualified in advanced life support skills (i.e., airway management, defibrillation, and use of resuscitative medications) and could promptly manage any adverse events that occurred during sedation. When the patient was fully awake and had no other abnormalities, the patient was transferred out of the postanesthesia care unit.

### Data collection

We collected the following data: age (< 65, ≥ 65), sex (male, female), body mass index (BMI) (< 18, 18–24, > 24), smoking and drinking, adjuvant therapy (no, chemotherapy, chemoradiotherapy), gastroscopy modality (unsedated, sedated), pathological tumor-node-metastasis (pTNM) stage (I-IV), pathology type (tubular adenocarcinoma, hypomucinous carcinoma, others), Borrmann type (0–4), tumor location (upper third, middle third, lower third), surgical resection range (distal, total, proximal), tumor diameter (< 5 cm, ≥ 5 cm), distant metastasis (no, yes), regional lymph node metastasis (no, yes), lymph node metastasis rate (percent of resected lymph nodes involved with metastatic cancer; <0.35, 0.35–0.74, >0.74), vascular invasion (no, yes), nerve invasion (no, yes), carcinoembryonic antigen (CEA) (< 5 ng/ml, ≥ 5 ng/ml), and carbohydrate antigen 199 (CA199) (< 37 U/ml, ≥ 37 U/ml). These variables were considered pathological risk factors that affected the survival of repeated GC patients and were analyzed as unfavorable candidate variables [20]. Pathologists performed pathological examinations based on the 8th edition of the American Joint Committee on Cancer Staging Manual [21]. In addition, due to the fact that patients undergoing sedated gastroscopies do not have exactly the same number of sedation cycles, we collected the number of sedated gastroscopies for each patient during the diagnosis and treatment process, and conducted subgroup analysis to further investigate the impact of sedated examinations on prognosis. Survival time was defined as the time interval between the date of gastrectomy and the date of death or the last follow-up, which ended May 31, 2021.

### Statistical analysis

All variables are count data and are described by frequency (percentage). Age, sex, history of smoking and drinking, adjuvant therapy, pathology type, tumor location, surgical resection range, tumor diameter, distant metastasis, regional lymph node metastasis, vascular infiltration, nerve infiltration, CEA, and CA199 were dichotomous or unordered multicategorical variables, and the two groups were compared using the χ^2^ test; BMI, pTNM stage, Borrmann type, and lymph node metastasis rate were ordered categorical variables, and the two groups were compared using the nonparametric Wilcoxon rank sum test. Survival curves were plotted using the Kaplan‒Meier method, and statistical analysis was performed using the log-rank test. Univariate analyses of each variable were performed using Cox proportional risk regression models to explore the factors that have an impact on survival. Variables that yielded *P* < 0.05 in univariate survival analysis were considered candidates in the multivariate Cox proportional risk regression model. Hazard ratio (HR) and 95% confidence interval (CI) were estimated for each factor. To reduce selection bias, propensity score matching (PSM) was performed to minimize confounding factors in the sedated gastroscopy group and unsedated gastroscopy group. The standard caliper width of the PSM used in this study was 0.02. Survival curves were plotted by GraphPad Prism 8. All statistics were analyzed using IBM SPSS Statistics version 26.0 software (IBM Corp., Armonk, NY). A value of *P* < 0.05 was considered statistically significant.

## Results

### Patient characteristics

**Fig. 1:**
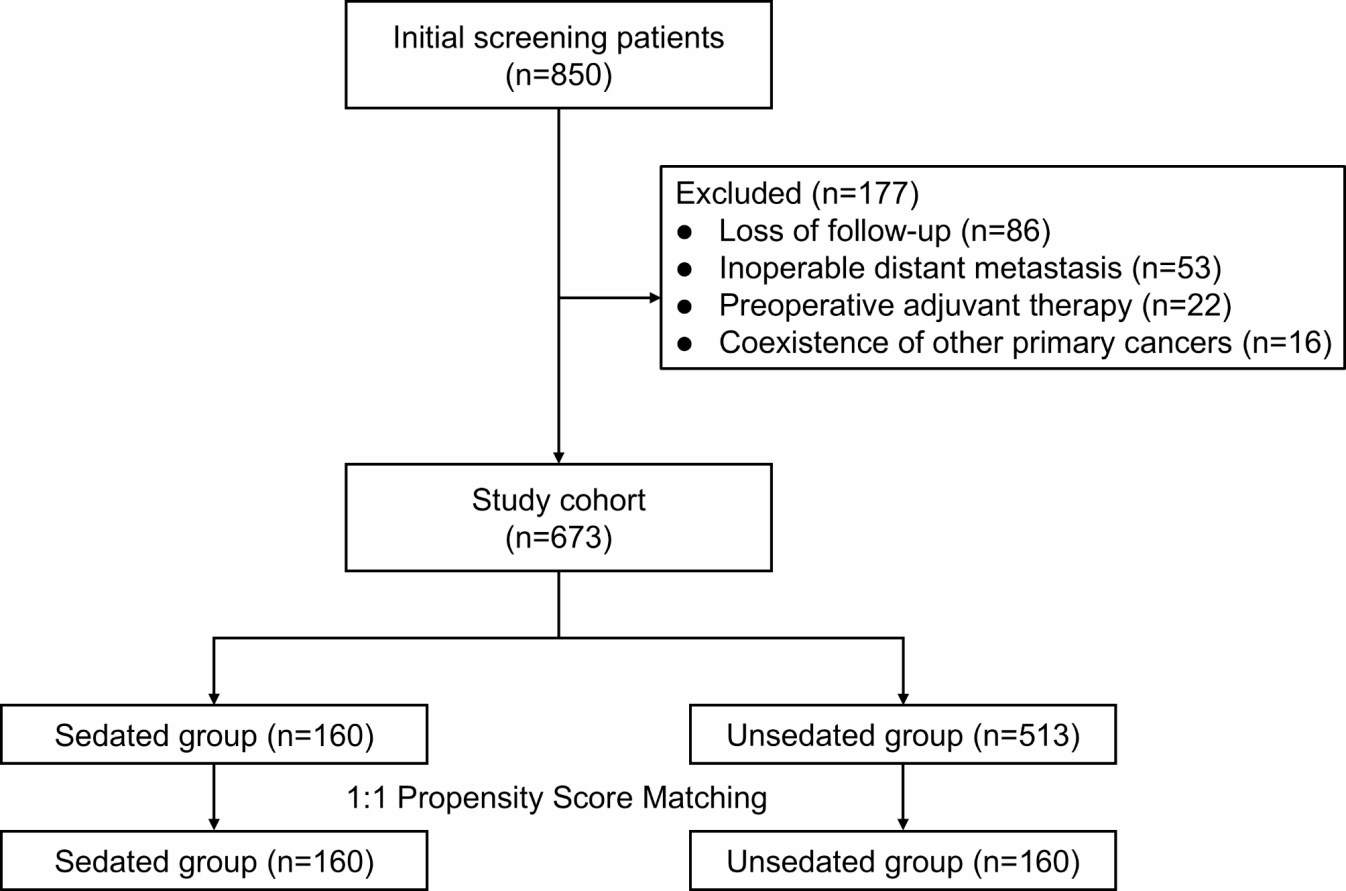
Flow chart of patient screening and exclusion criteria in retrospective analysis

During the study period, 850 patients were involved (Fig. 1). One hundred and seventy-seven patients were subsequently excluded from the analysis. Eighty-six of these patients were lost to follow-up, 53 patients were found to have inoperable distant metastases, 22 patients had preoperative adjuvant therapy, and 16 patients had coexistence of other primary cancers. Finally, the study included 673 cases eligible for analysis. Before PSM, there were no differences in age, sex, BMI, smoking and alcoholism, adjuvant therapy, pTNM stage, pathology type, tumor location, surgical resection range, tumor diameter, distant metastasis, regional lymph node metastasis, lymph node metastasis rate, vascular infiltration, nerve infiltration, CEA, CA199 between the two groups, but there was significant difference in borrmann type (*P* < 0.001, Table 1). After PSM was performed, 320 patients were matched at a ratio of 1:1, with 160 patients in each group. There were significant differences in vascular invasion (*P* < 0.001) and nerve invasion (*P* < 0.001) between the two groups, and there were no differences in the other variables.

**Table 1:** Basic and clinicopathologic characteristics of the patients before and after PSM

Variables	Overall patients			Matched patients		
	Sedated group	Unsedated group	*P* value	Sedated group	Unsedated group	*P* value
	(*n* = 160)	(*n* = 513)		(*n* = 160)	(*n* = 160)	
Age (y), n (%)			0.682			1.000
<65	130 (81.25%)	424 (82.65%)		130 (81.25%)	130 (81.25%)	
≥65	30 (18.75%)	89 (17.35%)		30 (18.75%)	30 (18.75%)	
Sex, n (%)			0.238			1.000
Male	106 (66.25%)	365 (71.15%)		106 (66.25%)	106 (66.25%)	
Female	54 (33.75%)	148 (28.85%)		54 (33.75%)	54 (33.75%)	
BMI (kg/m^2^), n (%)			0.901			0.781
<18	11 (6.87%)	30 (5.85%)		11 (6.87%)	10 (6.25%)	
18–24	93 (58.13%)	304 (59.26%)		93 (58.13%)	92 (57.50%)	
>24	56 (35.00%)	179 (34.89%)		56 (35.00%)	58 (36.25%)	
Smoking and alcoholism, n (%)			0.150			0.545
No	77 (48.12%)	218 (42.50%)		77 (48.12%)	68 (42.50%)	
Smoking	35 (21.88%)	155 (30.21%)		35 (21.88%)	42 (26.25%)	
Alcoholism	7 (4.38%)	30 (5.85%)		7 (4.38%)	11 (6.87%)	
Both have	41 (25.62%)	110 (21.44%)		41 (25.62%)	39 (24.38%)	
Adjuvant therapy, n (%)			0.891			0.436
No	27 (16.88%)	92 (17.93%)		27 (16.88%)	27 (16.88%)	
Chemotherapy	130 (81.25%)	409 (79.73%)		130 (81.25%)	126 (78.75%)	
Chemoradiotherapy	3 (1.87%)	12 (2.34%)		3 (1.87%)	7 (4.37%)	
pTNM stage, n (%)			0.746			0.432
I	59 (36.87%)	175 (34.11%)		59 (36.87%)	49 (30.62%)	
II	32 (2.00%)	122 (23.78%)		32 (2.00%)	43 (26.88%)	
III	68 (42.50%)	205 (39.96%)		68 (42.50%)	61 (38.12%)	
IV	1 (0.63%)	11 (2.15%)		1 (0.63%)	7 (4.38%)	
Pathology type, n (%)			0.618			0.272
Tubular adenocarcinoma						
Well differentiated	67 (41.88%)	217 (42.30%)		67 (41.88%)	61 (38.12%)	
Poorly differentiated	44 (27.50%)	149 (29.05%)		44 (27.50%)	62 (38.75%)	
Hypomucinous carcinoma	9 (5.62%)	19 (3.70%)		9 (5.62%)	7 (4.38%)	
Low-adhesion carcinoma	37 (23.12%)	124 (24.17%)		37 (23.12%)	27 (16.87%)	
Others	3 (1.88%)	4 (0.78%)		3 (1.88%)	3 (1.88%)	
Borrmann type, n (%)			< 0.001			1.000
0–2	86 (53.75%)	201 (39.18%)		86 (53.75%)	86 (53.75%)	
3	65 (40.63%)	260 (50.68%)		65 (40.63%)	65 (40.63%)	
4	9 (5.62%)	52 (10.14%)		9 (5.62%)	9 (5.62%)	
Tumor location, n (%)			0.493			0.293
Upper third	18 (11.25%)	42 (8.19%)		18 (11.25%)	12 (7.50%)	
Middle third	31 (19.38%)	104 (20.27%)		31 (19.38%)	25 (15.62%)	
Lower third	111 (69.37%)	367 (71.54%)		111 (69.37%)	123 (76.88%)	
Surgical resection range, n (%)			0.638			0.674
Distal	120 (75.00%)	389 (75.83%)		120 (75.00%)	125 (78.12%)	
Proximal	14 (8.75%)	34 (6.63%)		14 (8.75%)	10 (6.25%)	
Total	26 (16.25%)	90 (17.54%)		26 (16.25%)	25 (15.63%)	
Tumor diameter (cm), n (%)			0.848			0.808
< 5	112 (70.00%)	355 (69.20%)		112 (70.00%)	110 (68.75%)	
≥ 5	48 (30.00%)	158 (30.80%)		48 (30.00%)	50 (31.25%)	
Distant metastasis, n (%)			0.294			0.126
No	133 (83.12%)	407 (79.34%)		133 (83.12%)	122 (76.25%)	
Yes	27 (16.88%)	106 (20.66%)		27 (16.88%)	38 (23.75%)	
Regional lymph node metastasis, n (%)			0.995			0.911
No	72 (45.00%)	231 (45.03%)		72 (45.00%)	71 (44.37%)	
Yes	88 (55.00%)	282 (54.97%)		88 (55.00%)	89 (55.63%)	
Lymph node metastasis rate, n (%)			0.817			0.745
<0.35	138 (86.25%)	439 (85.58%)		138 (86.25%)	140 (87.50%)	
0.35–0.74	20 (12.50%)	65 (12.67%)		20 (12.50%)	18 (11.25%)	
>0.74	2 (1.25%)	9 (1.75%)		2 (1.25%)	2 (1.25%)	
Vascular infiltration, n (%)			0.213			< 0.001
No	101 (63.13%)	351 (68.42%)		101 (63.13%)	130 (81.25%)	
Yes	59 (36.87%)	162 (31.58%)		59 (36.87%)	30 (18.75%)	
Nerve infiltration, n (%)			0.978			< 0.001
No	89 (55.62%)	286 (55.75%)		89 (55.62%)	120 (75.00%)	
Yes	71 (44.38%)	227 (44.25%)		71 (44.38%)	40 (25.00%)	
CEA (ng/ml), n (%)			0.632			0.864
< 5	141 (88.12%)	459 (89.47%)		141 (88.12%)	140 (87.50%)	
≥ 5	19 (11.88%)	54 (10.53%)		19 (11.88%)	20 (12.50%)	
CA199 (U/ml), n (%)			0.235			0.070
< 37	148 (92.50%)	458 (89.28%)		148 (92.50%)	138 (86.25%)	
≥ 37	12 (7.50%)	55 (10.72%)		12 (7.50%)	22 (13.75%)	

PSM, propensity score matching; BMI, body mass index; pTNM, pathological tumor-node-metastasis; CEA, carcinoembryonic antigen; CA199, carbohydrate antigen199

### Survival outcomes

Firstly, we evaluated the incidence of distant metastasis between the sedated and unsedated gastroscopy groups during postoperative follow up. They had no significant difference in the incidence of distant metastases before PSM (16.9% vs. 20.7%, *P* = 0.294) or after PSM (16.9% vs. 23.8%, *P* = 0.126). (Table 1).

**Fig. 2:**
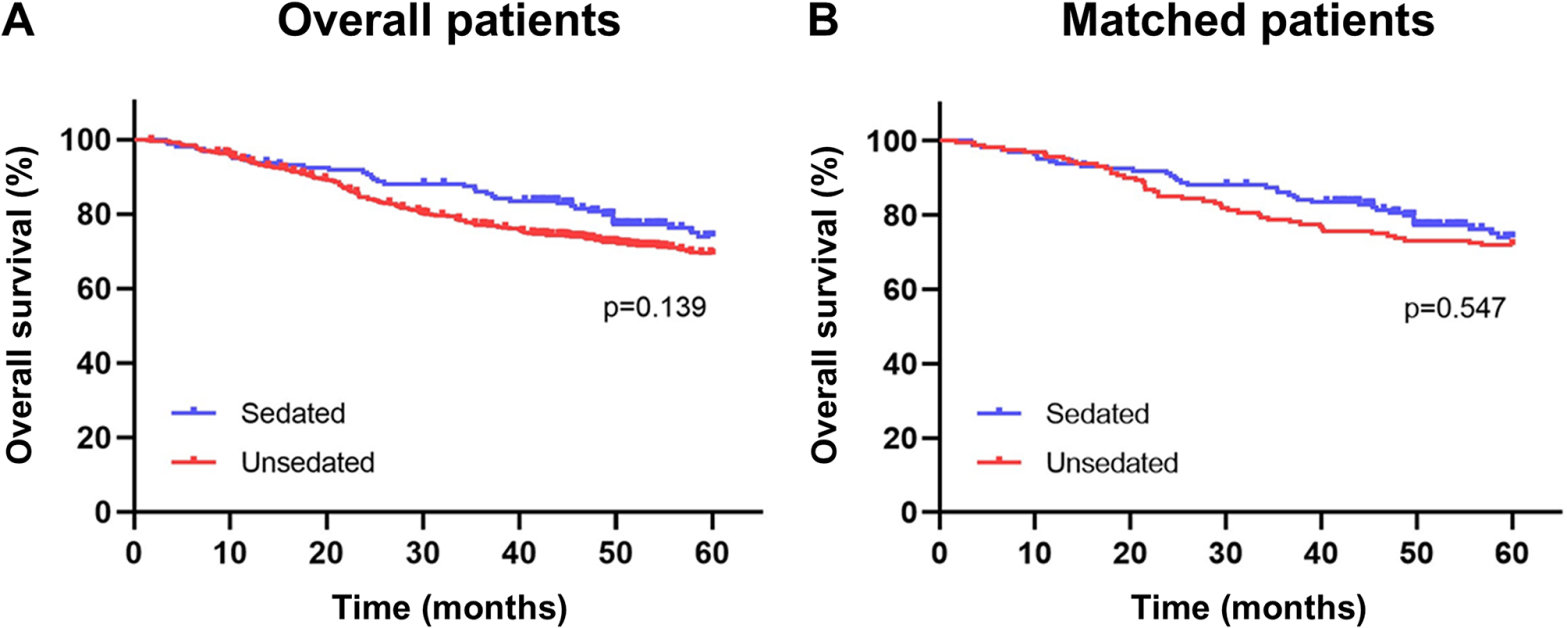
Kaplan-Meier survival curves of patients in both sedated gastroscopy and unsedated gastroscopy groups. **A**, overall patients before PSM. **B**, matched patients after PSM. PSM, propensity score matching

Second, we evaluated the survival outcomes between the sedated gastroscopy and unsedated gastroscopy groups. They had no difference in 5-year survival rate before PSM (73.9% vs. 69.4%, HR = 0.761, 95% CI: 0.531–1.091, *P* = 0.139) or after PSM (73.9% vs. 72.1%, HR = 0.874, 95% CI: 0.564–1.355, *P* = 0.547) (Fig. 2).

**Fig. 3:**
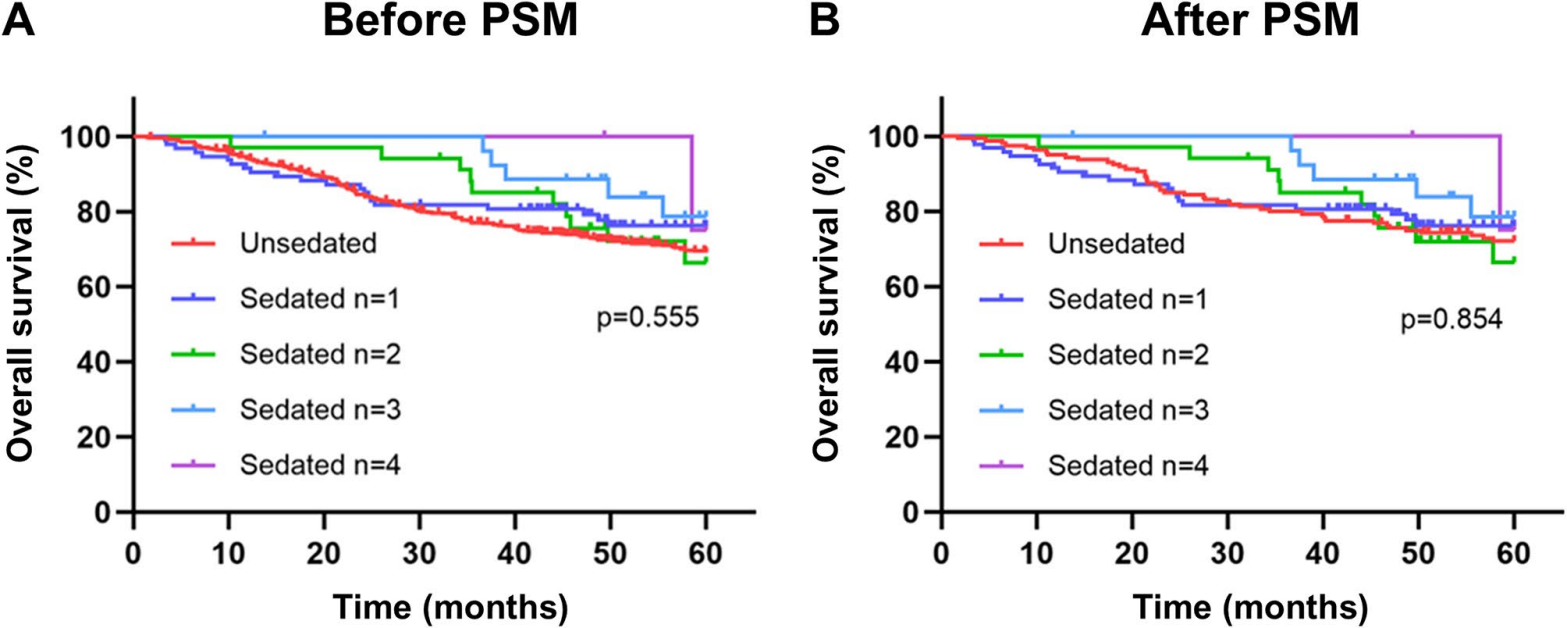
Kaplan-Meier survival curves for subgroup analysis of the number of examinations in the sedated gastroscopy group. **A**, overall patients before PSM. **B**, matched patients after PSM. PSM, propensity score matching

In the sedated gastroscopy group, the number of gastroscopy examinations done on each patient varied, up to a maximum of 4. Among them, 95 (59.4%) had 1, 33 (20.6%) had 2, 27 (16.9%) had 3 and 5 (3.1%) had 4 examinations. We also performed a subgroup analysis of the number of examinations in the sedated gastroscopy group of patients (Table 2). For 1 sedated examination, the HR was 0.768 (95% CI: 0.486–1.214, *P* = 0.258) before PSM and 0.882 (95% CI: 0.524–1.484, *P* = 0.636) after PSM. For 2 sedated examinations, the HR was 0.962 (95% CI: 0.507–1.826, *P* = 0.906) before PSM and 1.109 (95% CI: 0.558–2.206, *P* = 0.768) after PSM. For 3 sedated examinations, the HR was 0.555 (95% CI: 0.227–1.353, *P* = 0.195) before PSM and 0.634 (95% CI: 0.251-1.600, *P* = 0.335) after PSM. For 4 sedated examinations, the HR was 0.554 (95% CI: 0.077–3.958, *P* = 0.556) before PSM and 0.626 (95% CI: 0.086–4.546, *P* = 0.644) after PSM. The Kaplan‒Meier survival curves for the subgroup analysis are shown in Fig. 3.

**Table 2:** Subgroup analyses for the number of sedated gastroscopy examinations

Variables	Gastroscopy modality	Overall patients			Matched patients		
		HR	95% CI	*P* value	HR	95% CI	*P* value
Sedation times							
1	Unsedated	Ref			Ref		
	Sedated	0.768	0.486–1.214	0.258	0.882	0.524–1.484	0.636
2	Unsedated	Ref			Ref		
	Sedated	0.962	0.507–1.826	0.906	1.109	0.558–2.206	0.768
3	Unsedated	Ref			Ref		
	Sedated	0.555	0.227–1.353	0.195	0.634	0.251-1.600	0.335
4	Unsedated	Ref			Ref		
	Sedated	0.554	0.077–3.958	0.556	0.626	0.086–4.546	0.644

pTNM, pathological tumor-node-metastasis; HR, hazard ratio; CI, confidence interval

### Univariate and multivariate regression analyses

To determine the independent risk factors affecting the prognosis of patients after gastrectomy for GC, univariate and multivariate analyses based on Cox regression models were implemented. In the univariate Cox regression analysis, sex, adjuvant therapy, Borrmann type, pTNM stage, surgical resection range, tumor diameter, distant metastasis, regional lymph node metastasis, lymph node metastasis rate, vascular infiltration, nerve infiltration, CEA and CA199 were associated with overall survival (Table 3).

**Table 3:** Univariate Cox regression analysis of patient prognostic factors

Variables	Overall patients			Matched patients		
	HR	95% CI	*P* value	HR	95% CI	*P* value
Age (y)						
< 65	Ref			Ref		
≥ 65	1.407	0.994–1.992	0.054	1.358	0.804–2.294	0.253
Sex						
Male	Ref			Ref		
Female	0.571	0.402–0.810	0.002	0.704	0.434–1.140	0.154
BMI (kg/m^2^)						
<18	Ref			Ref		
18–24	0.670	0.396–1.133	0.135	1.071	0.425–2.694	0.885
>24	0.642	0.370–1.113	0.115	1.153	0.447–2.971	0.769
Smoking and alcoholism						
No	Ref			Ref		
Smoking	1.305	0.919–1.852	0.137	1.172	0.664–2.068	0.584
Alcoholism	1.517	0.840–2.740	0.167	1.287	0.502–3.304	0.600
Both have	1.330	0.913–1.938	0.137	1.543	0.915–2.604	0.104
Adjuvant therapy						
No	Ref			Ref		
Chemotherapy	0.660	0.467–0.934	0.019	0.465	0.283–0.764	0.003
Chemoradiotherapy	1.161	0.522–2.586	0.714	1.017	0.383–2.672	0.982
Gastroscopy modality						
Unsedated	Ref			Ref		
Sedated	0.761	0.531–1.091	0.139	0.874	0.564–1.355	0.547
pTNM stage						
I	Ref			Ref		
II	1.776	0.989–3.191	0.055	1.509	0.641–3.554	0.347
III	6.972	4.396–11.057	<0.001	5.622	2.867–11.025	<0.001
IV	7.606	3.232–17.898	<0.001	6.586	2.064–21.012	0.002
Pathology type						
Tubular adenocarcinoma						
Well differentiated	Ref			Ref		
Poorly differentiated	1.239	0.874–1.757	0.229	0.951	0.564–1.605	0.851
Hypomucinous carcinoma	1.261	0.631–2.521	0.512	0.995	0.352–2.812	0.992
Low-adhesion carcinoma	1.113	0.767–1.616	0.573	1.113	0.625–1.984	0.716
Others	1.027	0.252–4.186	0.971	1.274	0.305–5.320	0.740
Borrmann type						
0–2	Ref			Ref		
3	1.975	1.399–2.788	< 0.001	2.221	1.380–3.576	< 0.001
4	4.981	3.242–7.655	<0.001	3.957	1.922–8.149	< 0.001
Tumor location						
Upper third	Ref			Ref		
Middle third	0.889	0.499–1.583	0.690	1.200	0.417–3.454	0.735
Lower third	0.900	0.543–1.492	0.683	1.785	0.719–4.433	0.212
Surgical resection range						
Distal	Ref			Ref		
Proximal	1.485	0.852–2.587	0.163	0.889	0.356–2.219	0.801
Total	2.725	1.978–3.754	<0.001	1.980	1.188-3.300	0.009
Tumor diameter (cm)						
< 5	Ref			Ref		
≥ 5	2.086	1.560–2.789	<0.001	1.452	0.927–2.273	0.103
Distant metastasis						
No	Ref			Ref		
Yes	1.795	1.311–2.457	< 0.001	1.877	1.178–2.994	0.008
Regional lymph node metastasis						
No	Ref			Ref		
Yes	4.357	3.001–6.324	<0.001	3.816	2.207–6.596	<0.001
Lymph node metastasis rate						
<0.35	Ref			Ref		
0.35–0.74	3.825	2.769–5.284	<0.001	3.676	2.245–6.021	<0.001
>0.74	3.867	1.805–8.283	< 0.001	3.234	0.788–13.264	0.103
Vascular infiltration						
No	Ref			Ref		
Yes	2.455	1.838–3.281	<0.001	2.269	1.461–3.524	< 0.001
Nerve infiltration						
No	Ref			Ref		
Yes	2.613	1.934–3.530	<0.001	2.452	1.584–3.796	<0.001
CEA (ng/ml)						
< 5	Ref			Ref		
≥ 5	2.018	1.384–2.941	< 0.001	2.011	1.163–3.478	0.012
CA199 (U/ml)						
< 37	Ref			Ref		
≥ 37	2.085	1.424–3.054	< 0.001	2.8970	1.713–4.898	<0.001

HR, hazard ratio; CI, confidence interval; BMI, body mass index; pTNM, pathological tumor-node-metastasis; CEA, carcinoembryonic antigen; CA199, carbohydrate antigen199

Multivariate Cox regression analysis was performed on statistically significant variables from the univariate Cox regression analysis to further explore the factors affecting survival. In the multivariate Cox regression analysis, sex, adjuvant therapy, pTNM stage, Borrmann type and surgical resection range were associated with overall survival. Women had a lower overall mortality rate than men (HR = 0.557, 95% CI: 0.387–0.803, *P* = 0.002). Compared with pTNM stage I patients, the overall mortality rate of stage III (HR = 3.639, 95% CI: 1.722–7.693, *P* < 0.001) and stage IV (HR = 4.149, 95% CI: 1.459–11.801, *P* = 0.008) patients was increased. Compared with those who did not receive adjuvant therapy, the overall mortality rate was lower in those who received chemotherapy (HR = 0.393, 95% CI: 0.271–0.569, *P* < 0.001) and chemoradiotherapy (HR = 0.225, 95% CI: 0.093–0.545, *P* < 0.001). Compared with Borrmann type 0–2 patients, the overall mortality rate of type 4 (HR = 2.315, 95% CI: 1.426–3.759, *P* < 0.001) patients was higher. Compared with patients with distal gastrectomy, the overall mortality of total gastrectomy patients was higher (HR = 1.923, 95% CI: 1.351–2.737, *P* < 0.001) (Table 4).

**Table 4:** Multivariate Cox regression analysis of patient prognostic factors

Variables	Overall patients			Matched patients		
	HR	95% CI	*P* value	HR	95% CI	*P* value
Sex						
Male	Ref					
Female	0.557	0.387–0.803	0.002			
pTNM stage						
I	Ref			Ref		
II	1.485	0.770–2.865	0.238	1.101	0.424–2.859	0.844
III	3.639	1.722–7.693	< 0.001	2.244	0.721–6.990	0.163
IV	4.149	1.459–11.801	0.008	6.363	1.538–26.329	0.011
Adjuvant therapy						
No	Ref			Ref		
Chemotherapy	0.393	0.271–0.569	<0.001	0.217	0.124–0.377	<0.001
Chemoradiotherapy	0.225	0.093–0.545	< 0.001	0.180	0.058–0.561	0.003
Borrman type						
0–2	Ref			Ref		
3	1.223	0.841–1.780	0.292	1.245	0.719–2.156	0.433
4	2.315	1.426–3.759	< 0.001	1.588	0.652–3.867	0.309
Surgical resection range						
Distal	Ref			Ref		
Proximal	1.446	0.820–2.551	0.203	1.703	0.638–4.547	0.288
Total	1.923	1.351–2.737	< 0.001	1.741	0.978-3.100	0.060
Tumor diameter (cm)						
< 5	Ref					
≥ 5	0.912	0.655–1.271	0.587			
distant metastasis						
No	Ref			Ref		
Yes	1.310	0.925–1.857	0.129	1.271	0.756–2.136	0.366
Regional lymph node metastasis						
No	Ref			Ref		
Yes	1.375	0.770–2.456	0.282	1.667	0.653–4.259	0.285
Lymph node metastasis rate						
<0.35	Ref			Ref		
0.35–0.74	1.291	0.891–1.870	0.177	1.779	0.985–3.215	0.056
>0.74	1.526	0.677–3.441	0.308	1.054	0.213–5.210	0.949
Vascular infiltration						
No	Ref			Ref		
Yes	1.251	0.884–1.769	0.206	1.193	0.714–1.994	0.500
Nerve infiltration						
No	Ref			Ref		
Yes	1.102	0.761–1.597	0.607	1.349	0.797–2.2848	0.265
CEA (ng/ml)						
< 5	Ref			Ref		
≥ 5	1.311	0.881–1.951	0.182	1.579	0.844–2.954	0.153
CA199 (U/ml)						
< 37	Ref			Ref		
≥ 37	1.263	0.843–1.893	0.258	1.776	0.958–3.293	0.068

HR, hazard ratio; CI, confidence interval; BMI, body mass index; pTNM, pathological tumor-node-metastasis; CEA, carcinoembryonic antigen; CA199, carbohydrate antigen199

## Discussion

Our main finding was that sedated gastroscopy with propofol and unsedated gastroscopy were not associated with a difference in survival outcomes or distant metastasis after gastrectomy in patients with GC. The number of sedated gastroscopies was also not associated with survival outcomes. We evaluated other potential adverse factors to confirm that the survival of the patient population included in this study is consistent with that of GC patients in other studies. The results of the multivariate regression showed that sex, pTNM stage, Borrmann type, adjuvant therapy, and surgical resection range were all factors affecting survival.

In recent years, the number of patients opting for sedated gastroscopy has increased year by year as the demand for more comfortable medical care increases. Gastroscopy performed under sedation can reduce anxiety and improve patient satisfaction and comfort, increasing the willingness to repeat the examination [22]. Sedated gastroscopy increases patient tolerance, extends the possible examination time and allows endoscopists to examine multiple areas of the stomach more comprehensively, providing better results and reducing missed diagnoses [23]. However, in our study, the number of patients in the sedated gastroscopy group was only 160, much fewer than the unsedated gastroscopy group, at 513. A multicenter study showed that the majority of patients chose unsedated gastroscopy because they were concerned about the possible effects of sedated gastroscopy on cognitive function, distrust of the safety of the sedation procedure, and the high cost, all consistent with what we learned during follow-up [14]. A large multicenter registry study confirmed that the risk of major adverse events and sedation-related mortality in propofol-induced sedated gastroscopy was extremely low, and the mortality rate associated with sedation was only 0.004%, with a 0.01% risk of adverse events [24]. Several studies have shown that sedated gastroscopy is safe to use in high-risk populations, such as those with chronic hypertension and obesity and elderly individuals, and can reduce the incidence of adverse effects during examination [25–27]. Other studies have also shown that compared with traditional agents (midazolam, meperidine and/or fentanyl), propofol during gastrointestinal endoscopy results in faster recovery from sedation, discharge from the hospital and return to normal life, lower incidence of cardiopulmonary complications, higher quality of examination and better patient satisfaction [28]. There is also a risk of respiratory and circulatory depression during sedated gastroscopy examinations, depending on the dose of anesthesia and the depth of sedation, respiratory depression being the most serious complication, with an estimated 0.1% incidence requiring short-term ventilation with a face mask [29]. However, the probability of cardiopulmonary complications in patients in the sedated gastroscopy group during our study was low and was resolved promptly by the anesthesiologist. In summary, sedated gastroscopy has many advantages over unsedated gastroscopy: it can improve patient satisfaction and comfort, can be safely implemented, and is convenient for a better gastroscopy, so it is worth promoting.

The vast majority of current evidence on the effects of anesthetic techniques and anesthetics on long-term survival in cancer come from retrospective cohort studies. Compared to general anesthesia, epidural anesthesia did not improve the overall survival of patients with GC after surgery, but patients undergoing epidural combined with general anesthesia had fewer postoperative complications and lower rates of metastatic recurrence [30, 31]. As for local anesthesia, a multicenter randomized controlled trial showed that local infiltration with lidocaine improved overall survival and reduced metastasis in breast cancer patients [32]. Examining general anesthesia, a retrospective study that included 2,856 postoperative patients with GC found that patients in the propofol-based intravenous anesthesia group had better survival outcomes than those in the sevoflurane-based inhalational anesthesia group [8]. Another study agree, finding that total intravenous anesthesia was superior to inhalation anesthesia in terms of long-term survival outcomes for GC patients undergoing surgery [33]. This may be related to propofol’s inhibition of macrophage polarization, enhancement of the immune response and reduction of cancer cell invasion and angiogenesis [34, 35]. However, there have been conflicting results, as a large retrospective study of national databases failed to demonstrate any benefit in the long-term survival prognosis of propofol-based intravenous anesthesia for GC patients [36]. In addition, studies of liver and breast cancer found no difference in long-term survival between surgical patients who received total intravenous anesthesia and those who received inhalation anesthesia [37–39]. Laboratory evidence suggests that opioids may produce immunosuppressive effects and promote cancer cell development and metastasis [40]. However, one study found no difference in overall survival associated with perioperative opioid use in patients undergoing surgery for colorectal cancer [41]. Due to the low level of evidence from retrospective studies, there is no substantial evidence that regional anesthesia or propofol-based anesthesia improves long-term survival outcomes after cancer surgery, but several prospective randomized controlled trials are underway to determine the impact of anesthesia on cancer prognosis [42]. Stronger clinical evidence is still needed to validate the impact of anesthesia techniques and anesthetics on the prognosis of cancer patients and to guide anesthesiologists and cancer surgeons in their treatment choices.

In addition, each GC patient usually requires multiple gastroscopies during diagnosis and treatment. The smaller doses and shorter duration of anesthetic drugs used during sedated gastroscopy may not have a large impact on cancer suppression. However, patients with a preference for sedated gastroscopy will experience multiple sedations, and it remains unknown how a small number of multiple sedated gastroscopies will affect the long-term outcomes of cancer. We therefore also conducted subgroup analysis on the number of examinations performed on patients. Although the results showed no difference between the number of sedated gastroscopies and overall survival outcomes, the statistical robustness was inadequate to make a definitive conclusion. Therefore, according to the results of our study, each patient can individualize the choice of gastroscopy according to their situation and preference.

We conducted multiple regression analysis on potential adverse factors and obtained the following results. Women undergoing gastrectomy for GC had a lower overall mortality rate than men [43]. Regarding pTNM stage, patients with stage III and IV disease had an increased overall mortality rate compared to pTNM stage I patients [44]. Patients with Borrmann type 4 had worse survival outcomes than type 0–2 patients [45]. Patients who received chemotherapy and chemoradiotherapy after surgery had better survival outcomes [46]. The surgical approach to gastrectomy for GC can affect survival outcomes, with a higher overall mortality rate in patients undergoing total gastrectomy compared to patients undergoing distal gastrectomy [47].These conclusions are consistent with previous research findings, indicating that this study was divided into two groups based on sedated gastroscopy and unsedated gastroscopy, which showed similar performance to previous studies and achieved good PSM.

This is the first clinical study to examine whether sedated gastroscopy with propofol and unsedated gastroscopy have an impact on survival outcomes throughout the diagnosis and treatment of patients undergoing gastrectomy for GC. Our study has several limitations. First, this study was a single-center retrospective study, and the sample size of this study was based on the data available during the study period, with the possibility of insufficient sample size and statistical power. Second, we used PSM to adjust for differences in variables between the two groups, but there was still the possibility that the study was influenced by unmeasured confounders. Third, we only studied patients who underwent gastrectomy for GC, not patients who underwent other treatments without surgery, which may have resulted in incomplete coverage of the study population.

## Conclusions

There was no significant difference in survival outcomes or distant metastasis between sedated and unsedated gastroscopy in patients undergoing gastrectomy for GC.

## Data Availability

The datasets used and/or analyzed during the current study are available from the corresponding author on reasonable request.

## Abbreviations

GC: Gastric cancer
PSM: Propensity score matching
BMI: Body mass index
pTNM: Pathological tumor-node-metastasis
CEA: Carcinoembryonic antigen
CA199: Carbohydrate antigen 199
HR: Hazard ratio
95% CI: Confidence interval
